# Patients Submitted to Myocardial Revascularization with the Use of Bilateral Internal Thoracic Arteries: Diabetics *vs*. Non-Diabetics

**DOI:** 10.21470/1678-9741-2020-0292

**Published:** 2021

**Authors:** Achilles Abelaira Filho, Luis Ernesto Avanci, Thiago Faria Almeida, Rodolfo Witchtendahl, João Carlos Ferreira Leal

**Affiliations:** 1 Department of Cardiovascular Surgery, Hospital Sociedade Portuguesa de Beneficência de São José do Rio Preto, São José do Rio Preto, São Paulo, Brazil.; 2 Department of Cardiovascular Surgery, Faculdade Estadual de Medicina de São José do Rio Preto, São José do Rio Preto, São Paulo, Brazil.

**Keywords:** Mammary Arteries, Hospital Mortality, Cardiopulmonary Bypass, Operative Time, Coronary Artery Bypass, Myocardial Infarctation, Sternum, Diabetes Mellitus, Wound Infection

## Abstract

**Introduction:**

Use of bilateral internal thoracic artery (BITA) as graft in coronary artery bypass grafting (CABG) is controversial because it is related to higher in-hospital mortality, incidence of sternal wound-related infection, and an increase in surgical time. The primary objective of this study is to evaluate in-hospital mortality and mortality within 30 days from discharge. The secondary objective is to evaluate the occurrence of deep sternal wound infection in a 90-day follow-up.

**Methods:**

This is a retrospective review of the medical records of 152 patients undergoing elective CABG with the use of BITA and cardiopulmonary bypass (CPB). These patients were divided into two groups, diabetics and non-diabetics. Patients with acute myocardial infarction and concomitant valvular disease were not included in the sample.

**Results:**

Preoperative characteristics did not show significant differences between the groups, which allowed a comparative analysis. The variables electrocardiography time and aortic clamping time were higher in the diabetic group, with a significant statistical difference (*P*<0.0001). In-hospital mortality occurred in three patients, and there was no mortality up to 30 days in both groups. There was no significant difference in the primary end point between groups (*P*=0.56). Deep sternal wound infection was present in only one patient and showed no significant difference in the secondary outcome between groups (*P*=0.40).

**Conclusion:**

We did not observe a higher mortality and occurrence of deep sternal wound infection with the use of BITA in diabetic patients compared to non-diabetics, even with greater CPB and aortic clamping times in diabetics.

**Table:** 

Abbreviations, acronyms & symbols				
**AF**	**= Atrial fibrillation**		**G2**	**= Group 2**
**AKI**	**= Acute kidney injury**		**HBA1c**	**= Glycated hemoglobin**
**AMI**	**= Acute myocardial infarction**		**HDL**	**= High-density lipoprotein**
**BITA**	**= Bilateral internal thoracic artery**		**ICU**	**= Intensive care unit**
**BMI**	**= Body mass index**		**ITA**	**= Internal thoracic artery**
**CABG**	**= Coronary artery bypass grafting**		**LITA**	**= Left internal thoracic artery**
**CAD**	**= Coronary artery disease**		**LVEF**	**= Left ventricular ejection fraction**
**COPD**	**= Chronic obstructive pulmonary disease**		**SAH**	**= Systemic arterial hypertension**
**CPB**	**= Cardiopulmonary bypass**		**SAP**	**= Systolic arterial pressure**
**DAP**	**= Diastolic arterial pressure**		**SD**	**= Standard deviation**
**DM**	**= Diabetes mellitus**		**STEMI**	**= ST-segment elevation myocardial infarction**
**EuroSCORE II**	**= European System for Cardiac Operative Risk Evaluation II**		**TC**	**= Total cholesterol**
**G1**	**= Group 1**		**UA**	**= Unstable angina**

## INTRODUCTION

Coronary artery disease (CAD) is the most prevalent cardiovascular disease in the world, and it is higher in the population of patients with diabetes mellitus (DM)^[^^1^^]^. The risk of acute myocardial infarction (AMI) and sudden death in diabetics with CAD is similar to patients with prior AMI^[^^1^^]^.

In the United States of America, diabetics account for about 25% of the 1.5 million percutaneous and surgical interventions performed annually^[^^2^^]^. Coronary artery bypass grafting (CABG) plays an important role in the treatment of CAD, and recent studies show that CABG has superior results compared to other treatments in the medium and long-term outcomes, mainly in the subgroup of diabetics^[^^3^^]^.

The use of left internal thoracic artery (LITA) as a graft in CABG became gold standard after the publication of Floyd Loop, in 1986, being described for the anterior descending coronary artery, influenced by the reduction of risk of death, AMI, angina, reoperation, and a greater patency of 95% in a 10-year follow-up^[^^4^^]^. The study of a group from New York obtained a similar result, however, the follow-up was of up to 20 years^[^^5^^]^.

The superiority of LITA graft compared to venous graft (great saphenous vein) in relation to patency until hospital discharge was 98.0% *vs*. 91.7%, respectively, as demonstrated by angiographic studies. The analysis over 10 years after CABG showed that saphenous vein grafts are occluded around 50%, and, in 15 years, the saphenous vein was patent in only 30% of the cases compared to 90% of LITA^[^^6^^]^.

Recently, studies have demonstrated benefits of bilateral internal thoracic artery (BITA) use in diabetic patients^[^^7^^]^, and when stratified by ventricular dysfunction, the benefit was even greater. However, the use of BITA in diabetics leads to fears of infection and in-hospital mortality, which limits this practice^[^^8^^]^.

The primary objective this study is to evaluate in-hospital mortality and mortality within 30 days from discharge, and the secondary end point is the occurrence of deep sternal wound infection in diabetic *vs*. non-diabetic patients submitted to CABG.

## METHODS

This study is a retrospective review of the medical records of 152 patients undergoing elective CABG with the use of BITA and cardiopulmonary bypass (CPB) from February 2004 to April 2017 at the Hospital Beneficência Portuguesa of São José do Rio Preto, São Paulo/Brazil. The 152 patients were divided into two groups, diabetics and non-diabetics. In group 1 (G1) there were 61 (40%) patients with DM, and in group 2 (G2), 91 (60%) non-diabetic patients. DM was defined as fasting glycemia > 126 mg/dl in at least two measures or glycated hemoglobin (HBA1c) > 6.5%. For other risk factors, dyslipidemia was considered total cholesterol > 220 mg/dl, low-density lipoprotein > 140 mg/dl, triglycerides > 150 mg/dl, and high-density lipoprotein < 40 mg/dl; and systemic arterial hypertension (SAH) as systolic pressure > 140 mmHg or diastolic pressure > 90 mmHg. No obesity as body mass index (BMI) < 30 kg/m^2^, and obesity as BMI > 30 kg/m^2^. The European System for Cardiac Operative Risk Evaluation II (EuroSCORE II) score was calculated for all patients. Ejection fraction was analyzed with a 40% cutoff value for left ventricular dysfunction; atrial fibrillation (AF) was not present in the preoperative period. Patients with AMI and concomitant valvular disease were not included in the sample. Patients with prior AMI were not excluded from this sample.

The patients were operated by the same surgical team. Internal thoracic arteries (ITAs) were dissected in a skeletonized technique with the use of electrical bipolar scalpel and metallic clips. The right ITA was used as a free graft and, before CPB, it was anastomosed to the left thoracic artery in a Y-shape. The CPB was installed with arterial cannulation in the ascending aorta and the venous by the bicaval system, with total systemic heparinization according to the Bull protocol^[^^9^^]^. Cardioprotection used was Braile solution^[^^10^^]^. After the procedures, the patients were referred to the intensive care unit (ICU) for immediate postoperative care. The routine ICU stay was 48 hours. After hospital discharge, patients were guided to return and optimized medications. All patients underwent prophylactic antibiotic therapy with cefuroxime according to their weight. Glycemic control was performed to keep blood glucose taxes < 180 mg/dL, following the Society of Thoracic Surgeons guidelines^[^^11^^]^.

In-hospital mortality and mortality within 30 days from discharge were considered. After discharge, surgical wound was evaluated by the wound care team. Deep sternal wound infection has been defined according to the American Centers for Disease Control and Prevention definition^[^^12^^]^. It includes at least one of the following criteria within 90 days after the procedure: positive culture of mediastinal secretion obtained by puncture or from tissue fragment; evidence of deep sternal wound infection during surgical procedure; and fever, pain, or sternal instability associated with purulent secretion or positive blood culture.

This study was approved by local ethics committee (CAAE: 18235019.3.0000.8083), also complied with the Declaration of Helsinki.

### Statistical Analysis

For variables with homogeneous distribution, parametric tests were performed, and non-homogeneous tests were performed by non-parametric. Fischer's exact test was used to compare categorical variables and the analysis of quantitative variables was performed by the Mann-Whitney U test. Statistical analysis was performed by the StatsDirect Ltd. Stats Direct statistical software, England: StatsDirect Ltd. 2013

The preoperative characteristics of the two groups were analyzed based on the hypothesis test 0. Significant statistical difference was considered in the results with values of *P*<0.05.

## RESULTS

Among the 152 patients in the sample, the age range varied from 39 to 86 years (mean of 62.2 years), there was a higher prevalence of males (132), and they were divided into two groups - diabetics (G1: 61 patients) and non-diabetics (G2: 91 patients); we observed that in G1, with diabetic patients, only 12 (21.3%) were treated with oral hypoglycemic, and 49 (78.7%) were treated with insulin. HBA1c mean values were 7.65% in diabetics and 4.70% in non-diabetics, with statistical significance. HBA1c > 8% were found only in five patients, with no significance between both groups (P=1). Other risk factors: mean age in G1 was 62.09 years *vs*. 62.13 years in G2, there were 51 (83.60%) male patients in G1 *vs*. 80 (87.91%) in G2, and dyslipidemia was present in 38 (62.29%) G1 patients *vs*. 55 (60.43%) G2 patients, SAH in 52 (85.24%) G1 patients *vs*. 73 (80.21%) G2 patients, and obesity in 16 (26,22%) G1 patients *vs*. 19 (20,87%) G2 patients. Smoking was present in 20 (33%) G1 patients *vs*. 44 (48.35%) G2 patients. Left ventricular ejection fraction (LVEF) was < 40% in nine G1 patients (14.75%) *vs*. 15 (16.48%) G2 patients. It was diagnosed chronic obstructive pulmonary disease (COPD) in eight (13,11%) G1 patients *vs*. 14 (15,38%) G2 patients. Unstable angina occurred in eight (13,11%) G1 patients and 33 (36,26%) G2 patients, with statistically significance (*P*<0.05), prior STEMI in 23 (37,70%) G1 patients *vs*. 29 (31,86%) G2 patients. There were two patients on dialysis (3.27%) in G1 *vs*. none (0%) in G2. The calculated EuroSCORE II averaged 3.2% in G1 *vs*. 2.8% in G2 (Table 1).

**Table 1:** Patients' preoperative characteristics.

Preoperative characteristics	Total of patients (N=152)	Diabetics (N=61)	Non-diabetics (N=91)	*P*-value
Age (mean ± SD)	62.2±15.8	62±13.4	62.1±11.2	< 0.47
Males (%)	132 (85)	51 (84)	80 (88)	0.479
Dyslipidemia (TC > 240 mg/dl or HDL < 40 mg/dl)	93	38	55	0.866
SAH (SAP > 140 mmHg or DAP > 90 mmHg)	125	52	73	0.518
HBA1c % (mean)	152	7.65	4.70	0.004
HBA1c > 8%	5	5	0	1
Smoking (%)	64	20	44	0.066
LVEF% (< 40%)	24	9	15	0.824
Hemodialysis	2	2	0	0.159
No obesity (BMI < 30 Kg/m^2^)	117	45	72	0.44
Obesity (BMI > 30 Kg/m^2^)	35	16	19	0.44
COPD	22	8	14	0.8157
UA	41	8	33	0.0016
STEMI (prior)	52	23	29	0.4887
EuroSCORE II (average %)	3.6	3.2	2.8	0.999

BMI=body mass index; COPD=chronic obstructive pulmonary disease; DAP=diastolic arterial pressure; EuroSCORE II=European System for Cardiac Operative Risk Evaluation II; HBA1c=glycated hemoglobin; HDL=high-density lipoprotein; LVEF=left ventricular ejection fraction; SAH=systemic arterial hypertension; SAP=systolic arterial pressure; SD=standard deviation; STEMI=ST-segment elevation myocardial infarction; TC=total cholesterol; UA=unstable angina

All patients in the sample received BITA, however, other grafts were used when necessary, depending on the number of coronary arteries to be revascularized - 13 (8.55%) patients received radial artery, 129 (85%) patients received a great saphenous vein, and seven (11%) patients received radial and saphenous. Regarding the number of revascularized coronary arteries, 77 (50.65%) patients received four grafts, 53 (34.86%) patients received three grafts, and 17 (11.18%) patients had two grafts. In G1 (61 patients), the number of revascularized coronary arteries were two in five patients, three in 25 patients, and four in 31 patients *vs*. two in 12 patients, three in 32 patients, and four in 46 patients in G2 (91 patients). The mean CPB time and aortic clamping time in G1 were 105.90 minutes and 74.14 minutes, respectively, and 92.74 minutes and 67.3 minutes in G2. No patient had complex arrhythmia after CPB; however, up to six (9.83) G1 patients had paroxysmal AF *vs*. 11 (12.08%) at G2, all of them progressed to sinus rhythm after chemical cardioversion with amiodarone intravenously. Acute kidney injury (AKI) was observed in five patients (8,19%) in G1 *vs*. and seven patients (7,69%) in G2. None of the patients underwent prolonged mechanical ventilation (over 24 hours) (Table 2).

**Table 2:** Parameters during operation.

Parameters during operation	Total of patients (N=152)	Diabetics (N=61)	Non-diabetics (N=91)	*P*-value
CPB time (mean /min)	98	105.9	92.7	< 0.0001
Aortic clamping time (mean/min)	70	74.1	67.3	< 0.0001
Grafts				
Great saphenous vein	129	54	75	0.3611
Radial artery	13	4	9	0.5639
N. of coronaries revascularized				
Two	17	5	12	0.4352
Three	57	25	32	0.4975
Four	77	31	46	0.9999

CPB=cardiopulmonary bypass

In-hospital mortality occurred in three (1.9%) patients; two (3.27%) patients were in G1 (AKI and ventricular fibrillation) and one (1.09%) was in G2 (AKI), and mortality within 30 days from discharge was zero in both groups. There was no significant difference in the primary end point - in-hospital mortality and mortality within 30 days from discharge between G1 and G2 (*P*=0.56). Deep sternal wound infection was present in only one patient (1.63%). There was no significant difference in the secondary outcome, deep sternal wound infection, between G1 and G2 (*P*=0.40). Mechanical ventilation mean time was 630 minutes; in the diabetics group, the mean was 680 min and in the non-diabetics group, it was 635 min, and no significant difference was found between the two groups (*P*=0.9999) (Table 3).

**Table 3:** Postoperative parameters.

Postoperative parameters	Total of patients (N=152)	Diabetics (N=61)	Non-diabetics (N=91)	*P*-value
Atrial fibrillation	17	6	11	0.7954
AKI	12	5	7	0.9999
In-hospital mortality	3	2	1	0.9999
Mortality within 30 days from discharge	3	2	1	0.9999
Deep sternal wound infection	1	1	0	0.4013
Mechanical ventilation within 24 hours (mean/min.)	630	680	635	0.9999

AKI=acute kidney injury

## DISCUSSION

The use of BITA is controversial in the literature, although the use of two ITAs has shown a trend of better results^[^^8^^,^^25^^]^. Whenever possible we must use arterial grafts in CABG, this is a concept based on the greater patency of the arterial grafts when compared with venous grafts^[^^4^^,^^5^^]^. We know from the literature that 50% of the great saphenous vein grafts will be occluded over 10 years^[^^6^^]^, so that the use of ITAs as a graft becomes attractive for cardiovascular surgeons. ITAs have specific properties that prevent the presence of atheroma plaques in the intima layer, thrombus formation, and a lower degree of spasm. Undoubtedly, a greater patency over the years allows for increased survival and reduction of cardiac events, especially sudden death^[^^4^^]^.

In the last years, several studies have appeared comparing unilateral *vs*. bilateral ITAs with different subgroups^[^^8^^,^^13^^,^^14^^,^^18^^]^. In this study, we evaluated 152 patients with BITA divided into two groups, G1 (diabetic patients) and G2 (non-diabetic patients). Preoperative characteristics such as age, sex, dyslipidemia, hypertension, obesity, smoking, LVEF, and EuroSCORE II did not show significant differences between G1 and G2, which allowed a comparative analysis between groups. The percentual of males in this sample was high (85%), and this is an interesting point, but some studies in literature showed similar finds^[^^8^^]^.

In a survey conducted in the United Kingdom^[^^21^^]^ aimed at cardiovascular surgeons, 92% of respondents reported that use of BITA in CABG would likely or definitely be beneficial, 56% agreed that 25% to 50% of patients would benefit from CABG procedure involving the use of BITA, and 2/3 said that if they had to undergo a CABG they would like to receive BITA as a graft.

Non-insulin-dependent DM is an important risk factor for CAD progression, with 75% of these patients evolving to death due to cardiovascular complications. Studies about BITA^[^^22^^,^^23^^]^ showed prevalence of the insulin-dependent diabetic patients at 30% to 48%. This study showed a 78% insulin-dependent diabetic patients prevalence, with few presenting glycated hemoglobin > 8.0%.

CAD in a diabetic patient progresses more severely, with a higher number of coronary arteries involved and greater lesion complexity^[^^8^^,^^14^^,^^17^^]^. These characteristics make it difficult to handle anastomoses between grafts and coronary arteries, which prolongs operative time^[^^8^^,^^16^^]^. In our study, the time variables of CPB and aortic clamping were higher in the DM group with a significant statistical difference (*P*<0.0001).

In a recent meta-analysis^[^^19^^]^, Garcia evidenced the importance of complete myocardial revascularization as a better surgical strategy. Our study shows that the number of patients who received four grafts was similar in G1 and G2, 31 (50.81%) and 46 (50.54%), respectively, without significant statistical difference.

We know that AF is the most frequent arrhythmia in the postoperative period of CABG, affecting approximately 20% to 40% of patients. LaPar, in his study^[^^26^^]^, showed that DM was a predictive variable for AF in the postoperative period of CABG. In our sample, there was no statistically significant difference between the diabetic and non-diabetic groups.

The Arterial Revascularization Trial showed that the use of BITA did not increase the patients’ mortality in a five-year follow-up compared to the use of isolated LITA. In-hospital mortality and mortality within 30 days from discharge did not differ between G1 and G2 in our study^[^^8^^]^.

At present, BITAs are used in approximately 4% to 12% of CABG, and deep sternal wound infection is present in about 0.3% to 14% of these procedures. Taggart evidenced a twofold higher incidence of complications involving sternal wounding in patients undergoing BITA^[^^8^^]^. Lenz^[^^24^^]^ showed that the use of BITA was not associated with a significant higher morbidity. The strategy of preparing the mammary artery is very important to prevent endothelium hematoma and improve the sternal wound as well. Although some current studies demonstrate evidence of increased deep sternal wound infection in patients undergoing BITA, our study demonstrated that only one patient (1.63%) of G1 had deep sternal wound infection, and there was no significant difference between G1 and G2 (*P*=0.40). This result was probably obtained because only five patients had a glycated hemoglobin rate > 8%, in addition to the procedures being performed by the same team of surgeons using the same surgical technique.

Other factors such as low COPD prevalence, mechanical ventilation mean time, and ICU time may have influenced this result. The high incidence of insulin usage suggests advanced diabetes disease and more correlated problems such as higher infection rates, but it was also responsible for the good control of patients' blood glucose.

## CONCLUSION

The primary end point, in-hospital mortality and mortality within 30 days from discharge, was low, and the secondary end point, deep sternal wound infection with the use of BITA in diabetic patients compared to non-diabetic patients, was not statistically significant, even with greater CPB and aortic clamping times in the diabetic group.

We understand that the use of BITA in patients submitted to CABG should be more frequent.

**Table:** 

Authors' roles & responsibilities	
AAF	Substantial contributions to the conception or design of the work; or the acquisition, analysis, or interpretation of data for the work; drafting the work or revising it critically for important intellectual content; agreement to be accountable for all aspects of the work in ensuring that questions related to the accuracy or integrity of any part of the work are appropriately investigated and resolved; final approval of the version to be published
LEA	Substantial contributions to the conception or design of the work; or the acquisition, analysis, or interpretation of data for the work; drafting the work or revising it critically for important intellectual content; agreement to be accountable for all aspects of the work in ensuring that questions related to the accuracy or integrity of any part of the work are appropriately investigated and resolved; final approval of the version to be published
TFA	Substantial contributions to the conception or design of the work; or the acquisition, analysis, or interpretation of data for the work; drafting the work or revising it critically for important intellectual content; agreement to be accountable for all aspects of the work in ensuring that questions related to the accuracy or integrity of any part of the work are appropriately investigated and resolved; final approval of the version to be published
RW	Substantial contributions to the conception or design of the work; or the acquisition, analysis, or interpretation of data for the work; drafting the work or revising it critically for important intellectual content; agreement to be accountable for all aspects of the work in ensuring that questions related to the accuracy or integrity of any part of the work are appropriately investigated and resolved; final approval of the version to be published
JCFL	Substantial contributions to the conception or design of the work; or the acquisition, analysis, or interpretation of data for the work; drafting the work or revising it critically for important intellectual content; agreement to be accountable for all aspects of the work in ensuring that questions related to the accuracy or integrity of any part of the work are appropriately investigated and resolved; final approval of the version to be published
